# Exon Shuffling and Origin of Scorpion Venom Biodiversity

**DOI:** 10.3390/toxins9010010

**Published:** 2016-12-26

**Authors:** Xueli Wang, Bin Gao, Shunyi Zhu

**Affiliations:** Group of Peptide Biology and Evolution, State Key Laboratory of Integrated Management of Pest Insects & Rodents, Institute of Zoology, Chinese Academy of Sciences, 1 Beichen West Road, Chaoyang District, Beijing 100101, China; wangxueli@ioz.ac.cn (X.W.); gaob@ioz.ac.cn (B.G.)

**Keywords:** scorpion venom, molecular diversity, exon-intron structure, exon shuffling

## Abstract

Scorpion venom is a complex combinatorial library of peptides and proteins with multiple biological functions. A combination of transcriptomic and proteomic techniques has revealed its enormous molecular diversity, as identified by the presence of a large number of ion channel-targeted neurotoxins with different folds, membrane-active antimicrobial peptides, proteases, and protease inhibitors. Although the biodiversity of scorpion venom has long been known, how it arises remains unsolved. In this work, we analyzed the exon-intron structures of an array of scorpion venom protein-encoding genes and unexpectedly found that nearly all of these genes possess a phase-1 intron (one intron located between the first and second nucleotides of a codon) near the cleavage site of a signal sequence despite their mature peptides remarkably differ. This observation matches a theory of exon shuffling in the origin of new genes and suggests that recruitment of different folds into scorpion venom might be achieved via shuffling between body protein-coding genes and ancestral venom gland-specific genes that presumably contributed tissue-specific regulatory elements and secretory signal sequences.

## 1. Introduction

Scorpions are one of the most ancient arthropods and have existed for more than 430 million years without detectable morphological changes. Approximately 2000 species/subspecies occupy different ecological niches on Earth [1]. Under natural selection driven by predators and prey, they have developed an efficient venom arsenal for defense and predation. Scorpion venom is a complex mixture of peptides and proteins, most of which are neurotoxins adopting different folds. These toxins bind and modulate multiple ion channels (Na^+^, K^+^, Cl^−^, and Ca^2+^) in excitable and non-excitable tissues. In addition to these neurotoxins, some other components, such as antimicrobial peptides (AMPs), proteases, and protease inhibitors, were identified from scorpion venoms. Although the biodiversity of scorpion venom components has been well documented, its evolutionary origin is still enigmatic. It was previously assumed that many snake venom proteins might have originated via gene duplication of non-venom proteins expressed in the body [2] or pre-existing salivary proteins, which were subsequently recruited into the venom gland [3]. While this assumption appears attractive, a detailed mechanism with regard to how the recruitment event occurred remains unsolved. Among scorpion venom peptides, at least two classes of neurotoxins have been traced to ancestral immune-related body proteins, in which the short-chain toxins affecting K^+^ channels presumably originated from antibacterial defensins via the deletion of an n-loop to remove steric hindrance, and the long-chain toxins targeting Na^+^ channels from antifungal drosomycin-like molecules via evolutionary assembling of an NC-domain to capture a new site on the channels [4,5]. These observations highlight the convergence of the toxic origin in distantly-related venomous animals (i.e., from body proteins to venom proteins). Here, we come up with a new hypothesis about the origin of the scorpion venom diversity, in which exon shuffling between an ancestral venom gland-specific gene and a body protein-encoding gene is firstly proposed.

**Figure 1:**
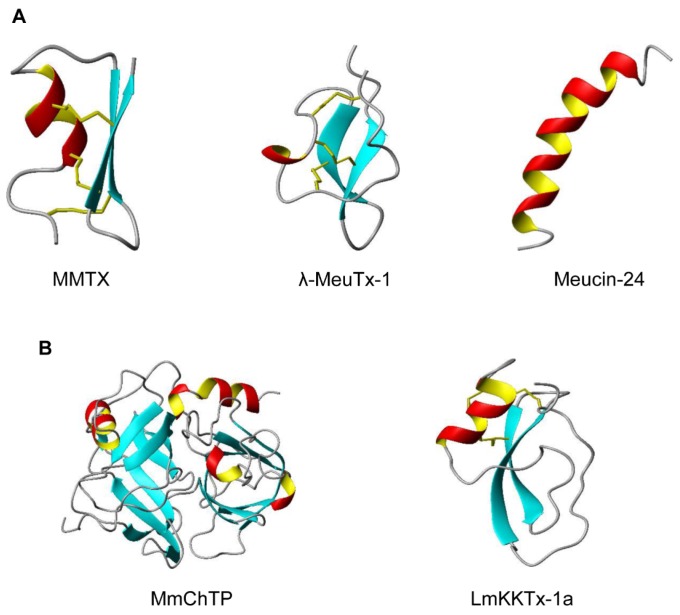
The fold diversity of scorpion venom components. (**A**) Representative structure of three different types of peptides: MMTX (PDB: 2RTZ) (CSαβ fold), λ-MeuTx-1 (ICK fold) [7], and the α-helical Meucin-24 (PDB: 2KFE); (**B**) Representative structures of scorpion venom-derived proteases and protease inhibitors: The chymotrypsin-like protease MmChTP whose structure was modelled on SWISS-MODEL (www.expasy.org) using the template of a mannose-binding lectin-associated serine proteinase-3 (PDB: 4KKD); the Kunitz-type protease inhibitor LmKTT-1a (PDB: 2M01).

Thanks to the whole genome sequencing of two scorpion species (*Mesobuthus martensii* and *Centruroides exilicauda*), it now becomes possible for us to analyze and compare more gene structures of scorpion venom components with different folds and functions. This will provide new insights into their origin and evolution. In this paper, we analyzed the gene structures of five representative scorpion venom components, including cysteine-stabilized α-helical and β-sheet (CSαβ) fold, inhibitor cysteine knot (ICK) fold, α-helical AMPs, proteases and protease inhibitors. Their representative structures are shown in Figure 1, which include the CSαβ-type MMTX (*Mesobuthus martensii*) [6], the ICK-type λ-MeuKTx-1 (*M. eupeus*) [7], α-helical Meucin-24 (*M. eupeus*) [8], the chymotrypsin-like protease MmChTP identified from the genome of *M. martensii* [9] and the Kunitz-type protease inhibitor LmKTT-1a from *Lychas mucronatus* [10]. We found that all genes encoding these proteins contain a common phase-1 intron located at the boundary between the signal and mature peptide-encoding regions. This discovery highlights a key role of exon shuffling in the recruitment of non-venom body proteins into scorpion venom.

## 2. Scorpion Venom Biodiversity

### 2.1. CSαβ-Type Peptides

The CSαβ fold contains an α-helix comprising the invariant motif (CX_[3]_C, X is any amino acid) that is connected to the second β-strand with another conserved motif (CX_[1]_C) by two disulfide bridges. The third disulfide bridge joins the N-terminus to the first β-strand [11]. Some CSαβ-type peptides have the fourth disulfide bridge in a variable position [12]. Scorpion venom-derived CSαβ-type peptides exhibit diverse biological activities, varying from neurotoxins targeting K^+^, Na^+^, Cl^−^, and Ca^2+^ channels to antibacterial defensins. It is known that the scorpion *M. martensii* genome encodes 116 venom neurotoxins, including 61 Na^+^ channel toxins, 46 K^+^ channel toxins, 5 Cl^−^ channel toxins, and 4 Ca^2+^ channel toxins [13]. Toxins targeting voltage-gated Na^+^ (Na_v_) and K^+^ channels (K_v_) are two of the most thoroughly studied scorpion venom components. The former contains 60–70 amino acids and 3–4 disulfide bridges; the latter consists of 23–64 residues and 3–4 disulfide bridges [14,15,16,17,18,19]. There are more than 300 scorpion Na_v_ channel toxin sequences deposited in the UniProtKB database (http://www.uniprot.org/). Based on different pharmacological features, these toxins are divided into two distinct classes, called α- and β-toxins [20,21]. The “Old World” scorpion toxins are mainly α-toxins, which cause a slowing of the inactivation process of sodium currents and a prolongation of the action potential by binding to receptor site 3 of the voltage-gated sodium channel [22,23]. The β-toxins are mainly from the “New World” scorpions, which cause the Na_v_ channels to shift the voltage dependence of activation to more negative membrane potentials and cause a reduction of peak current amplitude by binding to receptor site 4 [20]. More than 240 K^+^ channel toxins have been identified in scorpion venom (http://www.uniprot.org/), which are grouped into four major subfamilies (α-, β-, γ-, and κ-KTx) based on their sequence similarity and fold types. Apart from the κ-KTxs superfamily, whose members adopt a cysteine-stabilized helix-loop-helix (CSαα) fold, others are the members of the CSαβ superfamily. Several α-KTXs, such as ChTx [24] and MeuTXKα3 [25], also possess antimicrobial activity.

### 2.2. ICK-Type Peptides

In addition to the neurotoxins mentioned above, scorpion venom also contains peptides with an inhibitor cysteine knot (ICK) fold. To date, more than 15 such peptides have been described or deposited in the GenBank database (http://www.ncbi.nlm.nih.gov/) [1,7,26]. Of them, three have been structurally identified (λ-MK1a, imperatoxin A, and MCa) and five were functionally identified as either a K_v_ channel blocker (λ-MK1 and ImKTx) or Ca^2+^ release channel activators (imperatoxin A, MCa, and hadrucalcin). The remaining peptides are identified through screening scorpion venom gland cDNA libraries or analyzing transcriptomic and proteomic data [1,26]. The amino acid consensus sequence of scorpion venom ICK peptides was determined to be C_1_X_[6]_C_2_X_[4]_DC_3_C_4_X_[2–4]_K/RC_5_X_[3]_GX_[4–6]_C_6_K/R (X, any amino acid) with a ring size from 13 to 15 amino acids. The ICK structural motif is composed of an anti-parallel, triple-stranded β-sheet stabilized by a cystine knot where a ring is formed by two disulfide bridges (C_1_-C_4_ and C_2_-C_5_) and the interconnecting backbone, and the third disulfide bridge (C_3_-C_6_) crosses the ring, which is an especially stable structural motif [27]. Scorpion ICK peptides primarily target a limited number of receptors. For instance, λ-MeuKTx-1 isolated from *M. eupeus* is a blocker of the *Drosophila Shaker* K^+^ channel; Maurocalcine from *Scorpio maurus palmatus* and imperatoxin A from *Pandinus imperator* are the two most studied scorpion ICK peptides, which have been characterized as activators of sarcoplasmic reticulum Ca^2+^ release channels/ryanodine receptors of skeletal and cardiac muscles [28,29]. According to sequence similarity and phylogenetic analysis, scorpion ICK peptides can be divided into two distinct subgroups, which include the λ-KTx subgroup and the λ-KTx/calcine subgroup. Peptides within a subgroup are highly conserved, while peptides from different subgroups show greater divergence. Functional analysis indicates that these two subgroups of toxins can block K^+^ channels, but only he λ-KTx/calcine subgroups have the ability to activate Ca^2+^ release channels. Evolutionarily, all members in the λ-KTx subgroup belong to the Buthidae family, whereas all members in the λ-KTx/calcine subgroup are derived from non-Buthidae scorpions. These observations indicate that the divergence of these two subgroups might be associated with the speciation of scorpions [7].

### 2.3. Alpha-Helical AMPs

The scorpion venom gland communicates freely with the exterior via their open apertures at the end of telson and, hence, their glands could be contaminated by microorganisms present in prey and predators due to sting. In addition, some scorpion species often spray venom on their own bodies to clean them from dirty, and possibly saprophytic, organisms (bacteria and fungi), suggesting that the venom of these species might contain some sort of peptide antibiotics. Since the first discovery of Hadrurin in the venom of the scorpion *Hadrurus aztecus* [30], a growing number of α-helical AMPs are reported in other scorpion species [31,32,33,34,35]. These peptides are relatively small (2–5 kDa), cysteine-free, amphipathic, and basic molecules of variable length, sequence, and structure, considerably differing from scorpion toxins stabilized by disulfide bridges. They inhibit a wide range of microorganisms, including bacteria, fungi, protozoans, and viruses through the disruption of the membrane structure or/and cellular physiology function [36] and, thus, might play a role in the immune defense of scorpions against microbial infection.

### 2.4. Proteases and Protease Inhibitors

Previous studies have identified several proteases from scorpion venom, such as the chymotrypsin-like proteases ClP-1 and CIP-2 from the scorpion *Androctonus bicolor* [1]. In addition, using gene cloning techniques, Zhu and Gao isolated five cDNA clones encoding chymotrypsin-like proteases from the venom gland of *Mesobuthus eupeus* (GenBank accession numbers: ABR21038.1, ABR21039.1, ABR21040.1, ABR21070.1, and ABR21066.1). With respect to protease inhibitors, the first member was purified from the venom of the Indian red scorpion *Mesobuthus tamulus* in 1981 [37]. Subsequently, a Kunitz-type peptide Hg1 was identified from the Mexican scorpion *Hadrurus gertschi* by means of transcriptomic analysis [38], which is a trypsin inhibitor. Similarly, six other scorpion venom peptides (LmKTT-1a, LmKTT-1b, LmKTT-1c, BmKTT-1, BmKTT-2, and BmKTT-3) were also characterized as selective Kunitz-type trypsin inhibitors without activity against chymotrypsin and elastase but all having a potassium blocking activity. These Kunitz-type peptides usually have 50–70 residues cross-linked by two to four disulfide bridges [39]. Nearly all Kunitz-type peptides adopt a common structural fold comprising two antiparallel β-sheets and one or two helices [10]. In addition to these Kunitz-type trypsin inhibitors, scorpion venom also contains some Kunitz-type elastase inhibitors [40] and *Ascaris*-type serine protease inhibitors [41,42]. Although the abundance of these protease inhibitors is relatively low in scorpion venom, it is conceivable that they play a role in protecting toxins from degradation or serve as their synergistic factors.

## 3. Exon-Intron Structures of Scorpion Venom Gland-Expressed Genes

A eukaryotic gene is composed of exons that can be translated into proteins and introns that are non-coding DNA regions located between exons. Depending on their location relative to a codon, introns can be classified into three categories: phase-0 introns that exist between codons; phase-1 introns that are located between the first and second nucleotides of a codon; and phase-2 introns between the second and third nucleotides of a codon [43]. Studies have shown that the relative frequencies of these three classes of introns differ between genes encoding proteins with or without a signal peptide. For instance, as for the non-secretory human proteins, phase-0 introns were the most abundant (about 50%), phase-1 introns occupied 30%, and phase-2 introns were the least abundant (approximately 20%). However, in the secretory human proteins the frequency distribution of phase-1 introns (49.9%) were significantly more abundant than phase-0 introns (31.36%) and phase-2 introns (18.8%) and, importantly, phase-1 introns are enriched in the vicinity of the signal peptide cleavage sites [44,45]. This kind of biased distribution of phase-1 introns has been proposed as evidence in support of a possible role of exon shuffling in the evolution of signal peptides of human proteins. Similar to human secretory proteins, all the scorpion venom components mentioned here contain a signal peptide sequence directing their secretion to venom. Hence, analysis of their gene structures and intron phase distribution might help uncover the role of exon shuffling in their origin. The full-length amino acid sequences of scorpion venom protein precursors used in this analysis are provided in Material S1 of the Supplementary Materials.

### 3.1. Genes Encoding CSαβ Fold Peptides

**Figure 2:**
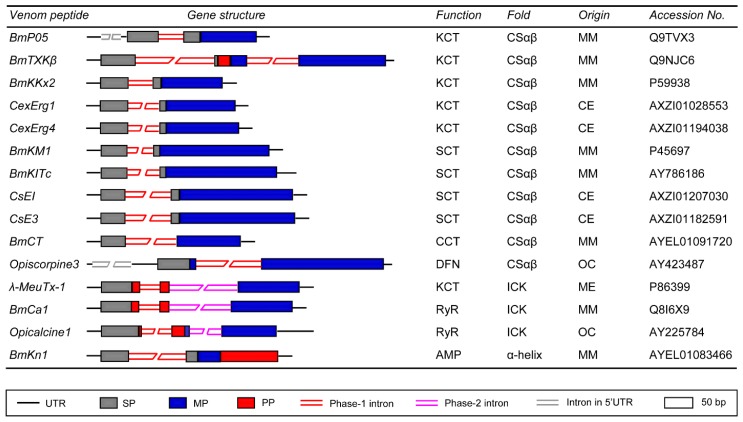
Representative gene structures of scorpion venom-derived neurotoxins and antimicrobial peptides. UTR, untranslated region; SP, signal peptide; MP, mature peptide; PP, propeptide. Functional classes: KCT, potassium channel toxin; SCT, sodium channel toxin; CCT, chloride channel toxin; DFN, defensin; RyR, ryanodine receptor; AMP, antimicrobial peptide. MM, *Mesobuthus martensii*; ME, *M. eupeus*; CE, *Centruroides exilicauda*; and OC, *Opistophthalmus carinatus*.

The gene structures of various CSαβ peptides from scorpion venoms are shown in Figure 2, which can be summarized as follows: (1) All of the members possess a phase-1 intron at the end of signal peptides in spite of the differences in the peptide length, pharmacological functions, intron sizes, and numbers; (2) In comparison with other members, the long-chain K^+^ channel toxin BmTXKβ has the second phase-1 intron located at its mature peptide-encoding region while the K^+^ channel toxin BmP05 and the defensin Opiscorpine3 have an additional intron compared to other genes, which is located at its 5′-untranslational region (5’-UTR) (Figure 2).

### 3.2. Genes Encoding ICK Fold Peptides

There are three scorpion venom ICK peptides whose gene structures have been determined. They are λ-MeuTx-1 from *M. eupeus* [7], BmCa1 from *M. martensii* [46] and Opicalcine1 from *O. carinatus* [47]. All these peptides share a conserved gene structure, as identified by the presence of a short phase-1 intron inside the propeptide adjacent the signal peptide and a long phase-2 intron at the N-terminus of the mature peptide (Figure 2).

### 3.3. Genes Encoding α-Helical AMPs

BmKn1 is an example of α-helical AMPs in the scorpion venom and its precursor is composed of three parts: a signal peptide followed by a mature peptide and a C-terminal propeptide. Similar to the peptides described above, its encoding gene has a phase-1 intron at the end of the signal peptide (Figure 2).

### 3.4. Genes Encoding Proteases and Protease Inhibitors

**Figure 3:**
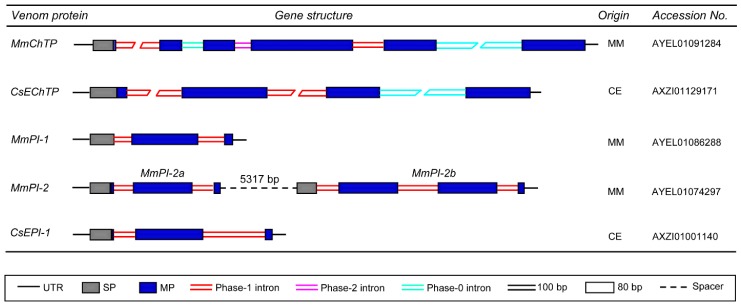
Representative gene structures of scorpion venom-derived proteases (MmChTP and CsEChTP) and protease inhibitors (MmPI-1, MmPI-2a, MmPI-2b, and CsEPI-1). MM, *M. martensii*; CE, *C. exilicauda*.

*MmChTP* and *CsEChTP* are two venom gland-expressed genes identified from the venom glands of *M. martensii* and *C. exilicauda*, respectively. They encode chymotrypsin-like proteases and have a relatively complex gene structure, in which multiple introns, including phase-0, -1, and -2, disrupt its mature peptide-encoding region. Despite this, these two genes also contain a phase-1 intron at the end of their signal peptides (Figure 3), as the case of other scorpion venom peptide-encoding genes (Figure 2). For the genes encoding the protease inhibitors MmPI-1, MmPI-2a, MmPI-2b, and CsEPI-1, they all also possess a phase-1 intron near the cleavage site of their signal sequences with one or two phase-1 introns in their mature peptide-encoding region (Figure 3).

## 4. Origin of Scorpion Venom Diversity by Exon Shuffling

Based on the presence of a common phase-1 intron at the end of signal peptides of CSαβ-type toxins affecting Na^+^, K^+^, and Cl^−^ channels, it was proposed that all of these components could originate from a common ancestor [48,49]. Subsequently, phase-1 introns were also found in a corresponding position of three cysteine-free scorpion venom peptide precursors and these were considered as evidence for common origin of these two classes of structurally-unrelated scorpion venom peptides [50]. However, our observation that the definitely evolutionarily unrelated scorpion venom components, such as linear α-helical AMPs, ICK-type toxins, proteases, and protease inhibitors, all possess such a phase-1 intron near the cleavage site of signal peptides indicates that this intron cannot be considered as evidence for divergent evolution of those components from a common ancestor. In fact, as mentioned previously, new structural and functional data derived from our experimental evolution have clearly demonstrated an independent origin of scorpion Na^+^ and K^+^ channel toxins from different ancestors despite their structural similarity [4,5]. Furthermore, it has been found that a phase-1 intron near the cleavage site of signal peptide is a universal feature of many secretory proteins [44], which is presumably due to a need of exon shuffling to create secretory capability.

**Figure 4:**
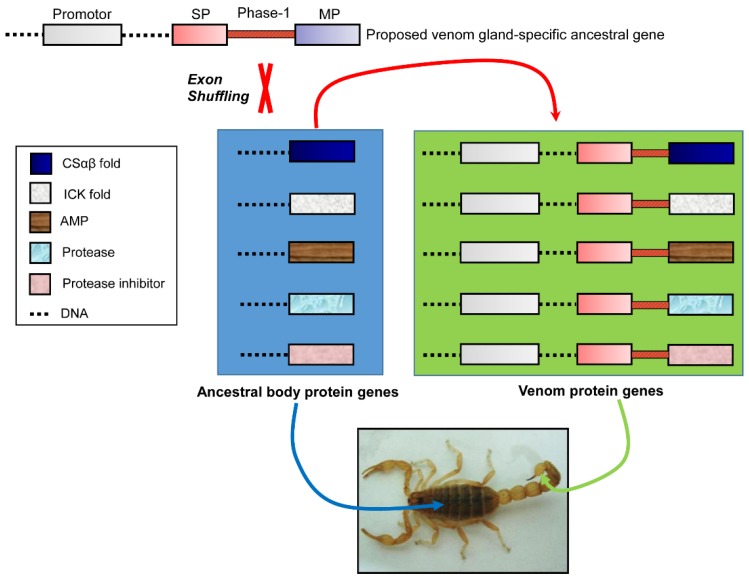
The hypothetical evolutionary model for the origin of scorpion venom biodiversity. Exon shuffling is proposed as a major evolutionary mechanism mediating the origin of venom proteins from ancestral body proteins, in which a venom gland-specific ancestral gene is considered as a donor providing two necessary elements for venom gland-specific expression: a promoter and a secretory signal.

The widespread distribution of a phase-1 intron at the end of a signal peptide hints its evolutionary relic character in developing a venom gland-specific secretory function. This matches a model of exon shuffling in creating a new gene by combining exons from unrelated genes [51]. We speculate that the first batch of ancestral genes earlier recruited into scorpion venom gland may be served as donors of exon shuffling to provide venom gland-specific signal sequences and promoter regions to non-venom body protein receptors (Figure 4), instead of multiple times of evolution of venom gland-specific regulatory elements and secretory signal sequences for each gene. Consistent with this speculation, two different scorpion venom peptide genes (*Opiscorpine* and *AaHI’*) have been found to contain a common venom gland-specific promoter module [52]. Likewise, similar modules were also observed in six additional scorpion toxin genes (*BmP05*, *BmKM1*, *BmKb1*, *BmCa1*, and *BmPI-1* from *M. martensii* and *CsEI* from *C. exilicauda*) (Figure S1, provided as Supplementary Materials; the promoter region sequences used in this analysis are provided in Material S2), suggesting a common regulatory role of these modules in controlling the gene expression of scorpion venom components. As a comparison, we also analyzed the promoter region of a *M. martensii* housekeeping gene (*β-actin*, ABV48915.1) that codes for a non-secretory protein expressed in non-venom tissue. As expected, the *β-actin* lacks several key C/EBPα transcription factor binding sites when compared with the venom gland-specific genes, in line with their differential tissue expression pattern. Apart from the case described above, exon shuffling was also assumed to evolve the function of invertebrate defensins and plant cytochrome c1 precursor, in which the exon encoding the mature invertebrate defensins was integrated downstream unrelated leader sequences during evolution [53], while plant cytochrome c1 precursor gained the mitochondrial targeting function from cytosolic glyceraldehyde-3-phosphate dehydrogenase [51]. It is noteworthy that these exon shuffling events represent a non-classical mode because, in classical exon shuffling, a proto-module exon often acts as a donor to be inserted between two protein-encoding exons (as a receptor) to form a larger mosaic protein [54]. However, in these examples, the donors are assumed to confer their signal sequences to the shuffled mature proteins (i.e., defensin, drosomycin, and cytochrome c1).

Our new observation regarding the gene structures of scorpion venom components reveals a genetic link in their regulatory and secretory regions rather than in the mature peptide part, presumably as a consequence of exon shuffling. In the subsequent evolution, gene duplication, followed by neofunctionalization driven by natural selection in a new environment (from body to venom gland), could further enlarge the biodiversity of scorpion venoms. A remarkable example in favor of our opinion is the finding that most of CSαβ toxin scaffolds evolved by episodic positive selection [55]. In addition to point mutations in a conserved toxic scaffold [56], structural adjustment of an old venom scaffold altering its fold type might also contribute to the diversity of scorpion venoms following exon shuffling, such as ICK and its derivative disulfide-directed hairpin (DDH) fold [27,55,57], and CSαβ and its derivative CSαα [58].

Given that scorpion and snake venom peptides/proteins were convergently derived from related body proteins, and both are secretory components, we surmise that exon shuffling might also be involved in the origin of snake venom. Analysis of gene structures of its components using the method described here will provide key evidence for this hypothesis. We believe that with more genomes sequenced, it is expected that new data will come out to support the commonality of exon shuffling in the origin of toxins from venomous animals.

## Supplementary Materials

The following are available online at www.mdpi.com/2072-6651/9/1/10/s1, Figure S1: Comparative promoter analysis of scorpion venom gland-expressed genes. Material S1: Sequences of Scorpion Venom Protein Precursors Used in This Paper. Material S2: Promoter sequences of scorpion venom gland-expressed genes.
