# Supplementary material for: Exon Shuffling and Origin of Scorpion Venom Biodiversity
Source: Toxins (Basel). 2016 Dec 26;9(1):10. doi: 10.3390/toxins9010010 (PMC5308243; doi:10.3390/toxins9010010)
Supplement: Supplementary file 1 [file toxins-09-00010-s001.pdf]

# Supplementary Materials: Exon Shuffling and Origin of Scorpion Venom Biodiversity

**Xueli Wang, Bin Gao and Shunyi Zhu**

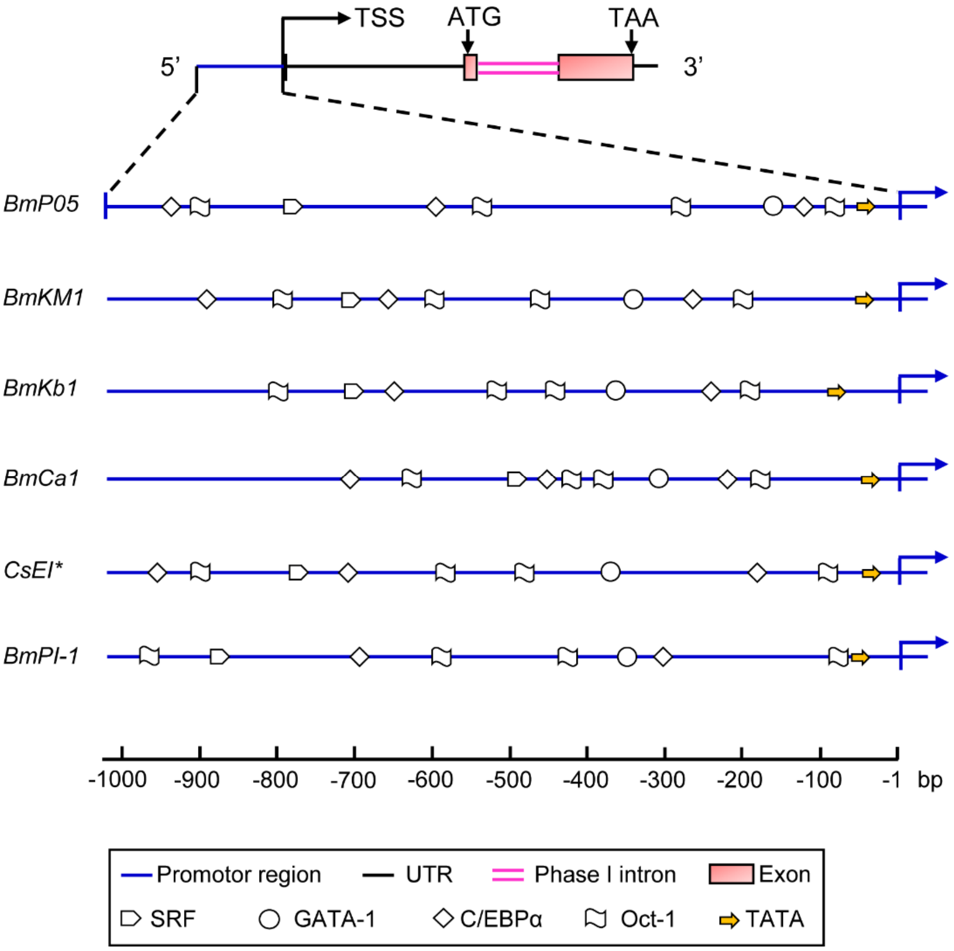


**Figure S1. Comparative promotor analysis of scorpion venom gland-expressed genes.** Promotor regions of *BmP05*, *BmKM1*, *BmKb1*, *BmCa1*, *BmPI-1* (*M. martensii*), and *CsEI* (*C. exilicauda*) were analyzed by PROMO [1]. Potential transcriptional factor (TF) binding sites were identified in the TRANSFAC database (http://www.gene-regulation.com/pub/databases.html#transfac). See Material S1 for sequence information.

**Material S1. Sequences of scorpion venom protein precursors used in this paper.** Signal peptides are shadowed in grey and propeptides in yellow. Amino acids whose codons are interrupted by a phase-1 intron or a phase-2 are bold in red or pink, respectively. The symbol (0) represents the position of a phase-0 intron. All the signal peptides were predicted in signalP 4.1 Server (http://www.cbs.dtu.dk/services/SignalP/) expect for CsEChTP, which was predicted in ProP 1.0 Server (http://www.cbs.dtu.dk/services/ProP/).

>gi|20140334|sp|Q9TVX3.1|**BmP05** *Mesobuthus martensii*

MHNYYKIVLIMVAFFAVI**I**TFSNIQVEGAVCNLKRCQLSCRSLGLLGKCIGDKCECVKHGK

>gi|57013104|sp|Q9NJC6.1|**BmTXKβ** *Mesobuthus martensii*

MMKQQFFLFLAVIVMISSVI**E**AGRGKEIMKNIKEKLTE**V**KDKMKHSWNKLTSMSEYACPVIEKWCEDHCAA

KKAIGKCEDTECKCLKLRK

>gi|38258408|sp|P59938.1|**BmKKx2** *Mesobuthus martensii*

MKISFVLLLTLFICSIGWSEARPTDIKCSASYQCFPVCKSRFGKTNGRCVNGLCDCF

>AXZI01028553.1|**CexErg1*** *Centruroides exilicauda*

MKVLILILIIASVMIMGVEMDRDSCDKSRCAKYGYYQECQDCCKKAGHNGGTCMFFKCKCA

>AXZI01194038.1|**CexErg4*** *Centruroides exilicauda*

MKVLILILIIASVMIMGVEMDRDSCVEKSKCGKYGYYGQCDECCKKAGDRAGTCVYYKCKCNP

>gi|6094252|sp|P45697.2|**BmKM1** *Mesobuthus martensii*

MNYLVMISFALLLMTGVESVRDAYIAKPHNCVYECARNEYCNDLCTKNGAKSGYCQWVGKYGNGCWCIE
LPDNVPIRVPGKCHR

>gi|59896057|gb|AY786186.1|**BmKITc** *Mesobuthus martensii*

MNYLVFFSLALLVMTGVESVRDGYIADDKNCAYFCGRNAYCDDECKKNGAESGYCQWAGVYGNACWCYK
LPDKVPIRVPGKCNGG

>AXZI01207030.1|**CsEI*** *Centruroides exilicauda*

MNSLLMITACLVLIGTVWAKDGYLVEKTGCKKTCYKLGENDFCNRECKWKHIGGSYGYCYFFGCYCEGLPD
STQTWPLPNKTCGKK

>AXZI01182591.1|**CsE3*** *Centruroides exilicauda*

MNSLLIIAACLALVGTVWAKEGYIVNYHTGCKYECFKLGDNDYCLRECKLRYGKGSGGYCYAFGCWCTHLY
EQAVVWPLPKKKCNGK

>gi|553808366|gb|AYEL01091720.1|**BmCT** *Mesobuthus martensii* Contig352952

MLLLFKSTLNILNSDPMCMPCFTTDPNMARKCRDCCGGYGKCFDPQCLCGYE

>gi|37594777|gb|AY423487.1|**Opiscorpine3** *Opistophthalmus carinatus*

MNNKLTALIFLGLLAIASCKWLNEKSIQNKIDEKIGKNFLGGMAKAVVHKLAKNEFMCVANVDMTKSCDT
HCQKASGEKGYCHGTKCKCGVPLSY

>gi|332278124|sp|P86399.2|**λ-MeuTx-1** *Mesobuthus eupeus*

MSTFIVVFLLLTAILCHAEHAIDETARGCNRLNKKCNSDADCCRYGERCISTGVNYYCRPDFGP

>gi|122069910|sp|Q8I6X9.2|**BmCa1** *Mesobuthus martensii*

MNTFVVVFLLLTAILCHAEHALDETARGCNRLNKKCNSDGDCCRYGERCISTGVNYYCRPDFGP

>gi|37539455|gb|AY225784.1|**Opicalcine1** *Opistophthalmus carinatus*

MKPSLIIVTFIVVFMAISCVAADDEQETWIEKRGDCLPHLKRCKENNDCCSKKCKRRGTNPEKRCR

>gi|553816961|gb|AYEL01083466.1|**BmKn1** *Mesobuthus martensii* Contig343431

MKSQTFFLLFLVVLLLAISQSEAFIGAVAGLLSKIFGKRSMRDMDTMKYLYDPSLSAADLKTLQKLMENY

>gi|553808811|gb|AYEL01091284.1|**chymotrypsin-like protease (MmChTP)** *Mesobuthus martensii* Contig352514

MSSIFNVVFLSLLVSSILYFKLEDEKRIYGGRYANPGEFPWM(0)VFIKVTDELNCSGYLISSSYILTAAHCMISANI
FNSIFSLFSPLQDMTARIGNIDSDSGQEYTFQSFSKHPDYDNSTFYGDIALLKLSTPVTFTPHVNRICLPSNNAFY
NHETPVLQMGWGRFSNTSEQVTKILKVTDIGKVYGHNECQQLFDSLNITLPNGHVCVKNSGIDGVCE(0)GD
SGGPLVVRRGTEYTAIGSDSVGFYANCSVDNNFVEIFNDIFYHRQWIIDQMDESICEN

>AXZI01129171.1|**chymotrypsin-like protease (CsEChTP)** *Centruroides exilicauda*

MILRILLTGELYWVLEEPELETTRALETVNSKENKMWAKIGTVDREEGQEYRFRSSRVHPDYSNLTYHGDIALL
KLTSPVVFDRNIDRICLANDRNYYRGNTPVLQMGWGRFSNESAEVTRILKVTEEGYIFDHGDCSDMFAFFNY
TLWDGTVCIKNSGSEGVCE(0)GDSGGPLVTRNGNSYTAIGLESIGFYENCTVDNSFAEVFTDLLYHRQWIVDN
VDETICQQ

>gi|553814002|gb|AYEL01086288.1|**protease inhibitor-1 (MmPI-1)** *Mesobuthus martensii* Contig346835

MSSLQILCLVFALNIVISIAHSKHGSINCRLPPERGPCRGNITKYYYHNESRTCRTFSYGGCEGNSNNFRNRHY

CMKYCARKRHGWLGTGWI

>gi|553826262|gb|AYEL01074297.1|**protease inhibitor-2a (MmPI-2a)** *Mesobuthus martensii* Contig331538

MKWILVVCVVFSFFNFYFGEEEDCCSYEVDPGPCFARFDRYFYNTANKNCEHFIYGGCKGNCNNFNNQSEC

CENCGGNNCGED

>gi|553826262|gb|AYEL01074297.1|**protease inhibitor-2b (MmPI-2b)** *Mesobuthus martensii* Contig331538

MKWILVILVIFSLFNFYLKDCCNYKVNPGPCFALIPRYFYNRISKNCEHFSYGGCKGNCNNFYNQSECCENCG

GKDCCNYKVNPGPCFALIPRYFYNRISKNCEHFSYGGCKGNCNNFYNQSECCENCGGNNCNEY

>AXZI01001140.1|**protease inhibitor-1 (CsEPI-1)** *Centruroides exilicauda*

MKSSTILYLVLILNILLVSAKHHGRINCRLAPKKGSCNDRIMKWYYNSKNKTCEAFIYSGCDGNTNNFRNKH

NCLKYCVRKRHGFGVFDLS

**Material S2: Promotor sequences of scorpion venom gland-expressed genes** (*BmKb1*, *BmP05*, *BmKM1*, *BmCa1*, *CsEI* and *BmPI-1*). Transcription start site (TSS) are boldfaced in red. The predicted transcription factor (TF) binding site of SRF, GATA-1, C/EBPα, Oct-1 and TATA are shadowed in cyan, green, yellow, pink and blue, respectively.

>BmP05

TTGAGAATATCCAATTTAGAGTTTGCAATATCTGCAGAGGAAAGAAATATTGTGCATTATTCTTTTTGCCC
GGGGTACCCGGGCAAAGTGGATTTTCAATTAGCGCAAAGTGAATAACACATGTAATCGAACTAGCAGA
TTATTTGCTGATGGAATGTATGCCTCGCCACAGATGGCAGCGTGTCTCGCCTTTAGCAAGCTTTTTTAGTA
AATATATACATATTTTCTTAGACTACGATATAGAGATCAACTAACACGAATCATTGGTTTAAAGATTCGTG
TTAGTCGATCTCTATATCGTGTTCTAAGAATGTATGTATATATTCATTAGGAAAACTTGCTAAAGGCGACG
CGACACGTCTTTCATTAGCAGGTAATCTACCATTTTGTCGAAACCTTCTTTAAAACAAATTAATTAACAC
TCAACACACAAGAAAATCATATTTGAAACTAACGCACATCTCATTAGCAATATGTGATCAGTACAGGAA
CCATGACTAGAACTCAGAGACAAATTTAAATTTGCATAAAATCCTCTGATAATGCATGTTTCGTATAACG
CGAAACGTATCCCTCGCGTTGTACGAGAATCCGCCGTATAATAATTAATTTTACTTTTTATGATAAACATA
TAAATTTCTCATTTTTTTAAATAAAATAGATTCTATTTTTTGGTGATTACGAAATATTTTTCATTTCCTAGAA
TCCGCTTAAACTAAAATTTCACAGAGTATAAAGTTTAATTTTGGGGAGAGGGAAAAAGTTGATGTGTTC
TGTAAGATTAGTTTTATAAATAATAAAGATAAACTAATAAATGCTTACAGAATTAATATTTCTAATGTTTA
ATTTTATGGCTAGAAGTTTTGTTAATATATGAAAGTTTTAATTGCATCCCATTAAGTTTTAATGTTTCTTCCT
GCAATGTGTGCTATTTTATGTCATTTTAGCTGAGAACAAATTGTATATAAACTGCAGCTATTTCTATTCTTA
ATATAATTCC**A**

>BmKM1

TAAAATTCGGAAAAATCGCGTTGTATTGAAACAGCGTTAAGCGGGGTCTTACTGAATGTAAAGAAGGC
TGTATTTTAATATCAATCCAGTCCTTTCAGCACATTATAATGTTCGGGGGACCCGAACATTAAAACACGA
TCACTATGACCTCTGTTTCTATAACTTATAAAATACGTATTATACAGTAGCAATGCAGAATTGTATATTTTT
TATTTAAAATCTGAAAAGGATATGTATGTTTATGATATTCAAACTTCTTCTGCATTATTGGGGCATATTCTC
ACTTCGTACTGTTTTATGATATTTTTTTTATTTAAAGAGAATTTTATATTCAATTAAACAAACAAATCTCTT
CCTTATGAGTAATAACGATGGTGCGATAATTACATTTGCTGTAATTATTAAATGGTATTTAATTTATCAGAT
AAAGAAATTACGTATTCGGAAAACTCCATTTATTGCCATTCCTTCTCCATCCTACTCATCCTGGCGTCAC
TAATGTATGTTTATTCGAAGATGGCGTGACAATACTAAATGATATTTAAATTTACACAAGACAATATTATA
TGCAAAATTATCCGTTTGTGCTAATTTTAAAGCAAATCTTTGTTTAATTTTATATTTTTTATGTTGGAAGA
TTTGTTGACAATTATCCACACAAATATTGAAGAAATTAATCGGTAAAACAAATTTATCACATTGATTTAA

TTAATAAATTTACATTTGGCATAAAAGTAAATTTCTTTATTTGTAATTGCATTTAGCAATTATTAACTGTCG
ATGATAGGGATATCCATTTTCAATAAGAGTCGATAATAAAACGACTTGTGCCATCTGCATTCTGTATCCA
GATTGGTCTGTTGTTTATTATTTCCTTGTCATTGGCTGATAGCATTCGCCTATTGGTAAATTTATTAACTATT
AAAATAATTGCTGTTGTGGGTGTTCCCTTTCTGTGCTATATAAACAGCGATTTCATTGTCCAAAGGCAAC
AATTCA

>BmKb1

TTCGAGTTTGGTGTTGGACGGATGGTGGAGACCAGGATCGACGGGCCGGGCCTGGGCCCTATGATAAA
TATTTTCATGGGGTGGGAGCCGAGACTCGAACCCGGATCCTCCAGAATGAAAAGCCGAAGAGTTTCCA
CGCGACTAATCCGGTCGACTTTGGAATCGATTATTAACGCCCAGTAGAATGGATTAACTGCTCAATCGC
CTACTCAATGTTAGAAAAACGAGGACATCATTTGGGTTGGCTATTTTCTCGAACGTACATTCAGTTCTAT
AATAAATTGACTTTGTTTTGGTGGCCTGACATATCCCATTAAAACAAAGTCAATTAATTACAGAATTGAA
TGTACGTTCGCGAAAATAGCCAATCAAACGACATTCTCGTTTTTCTAACATTGAGCGGTCAATCCCGCA
ATACAGCATGATGATTGTCATATTTTTTTCTCTAAGTGCCAAAGATGGGCATGCCTAAAATGTCGCATTTA
TAAATTTCGGCCGTTTGAGCGACTTTTTTTTTGGCCATTTTCTAAGTAACGCTTAGCAGCCTCTGGAAAAT
ATTAATTCGATATATAATTACAAATTATATATTTTGAAAAAATTTTCGTATTTTATAATTTGTCGAAAATTTA
TTCGTTAATTAGTTTAAGGCCATAACCGTAGAAGAGTGGTAAGGGCCGAGTTGAAAATACGCATTAAAA
TGTCGGATAATACGTAATTATAAAACGTCGGCGGACAACATATTTTTTACATTTTAAAAAATAACAATTG
TAAAACGAATAATTGACGTTTTTCA

>BmCa1

GGCCCGTTGAACCCCACCTTCAACCATCCGGTTGATCCCACCATGGATGTTGGCCAGCTTGTGATGGGC
TCTATAAATGGCCGCCGCCAGATACATATACCCTTCGTACTATATGGCGGCTCAACTAACGTAACTTAAC
TGGTCAATAATTATGGTAGGATCAACCGGAATCTTGAAAAATGGAATTCGACGAGGCGAGTCTTGACCC
ATTCTGAAAATTACAGGTGTGGAACCCGGATCTGAACCCAGGATCCATCGTCGGCCGGATTGGCCGCG
TGGAAACGCTTCGGCTTTCTACACTGGAGGACTCGGGTTCGAATCCCGGGTCCCCCCTTGAAAATATTT
ATCATGGAGTCCGGGCCTGGCCCATTGATCCCTGTCCTCCGCAATCCGGCTGACCCCTACGCAGGGATG
GGCAGTCTGTGGAACGGTTTGTTTCGCCCCGCCACATAGTATTCTACCTTAATGGAGTGTGGCGTCATCT
GTTAACAAAAAACCCAGGATCCATCGTCGGATTGCTGTCATCCGGTCAAAAGAAAAAAGACATTTGGA
GAGAAAATTCATTAAAATATACAATAACCAAATTTTCCTTTTGTAGTATACTATAAGAATTCATATTGGTA
ATAAACATACAGTACCTAATAAATTTCATTGTGAATAATTTCTTAAGTGGCCATAAATAATATTTATCTTTC
AATGTGAAATGTTAAATATTAAGAATAATCTTCCTATAATATTTTTTATTTAAAAATAAATATGAAATAATG
AAAATTTACGTTGAAAACATGTCTGGTTTTTAATTTTATTGCTATAAGCATACGGAAAAATTATATTTAAT
ATTCTACAAACTAGAATTGTAAAAGGTCACGCCGACTTAATAACTTGAAACAGTTGTTTTCTGATATTAA
AATTTATAAAATAAATATAATAATTGTTTTAAATAAATGGAAGAAAATATAATTGTGAATTATATAAATGA
AAAACCATTTGTTGAATTTGGA

>CsEI

CAAAATCATAACAAAAAAGTCAAGAAACGTTTAAACAAAATGGTTGTAAATATTCTGTAATTTTCAATT
ATACAACGATATAAAATTAAAATACTAGCAAAACACTAAAGAAAATAATTTTGATGAAATTCCATAAAA
AATACTTAAATTTAAGAAACGCTAATACGCATTTTCTCAACGTTATAATGTGCGTAGCTCCACCTCTTAC
GAGATTTTACTAACAACTCATTTTTATATTACTAATTATTACAAAGAGATATAATTTTTCAAAAAAAAAAG
AAATTTTTTAAAATATTGCAAATGCCAATCAAAAACAAAAATTTTCATTAAGTTCATCTTTACTCGAGAT

ATATAGCCTATTTAACATATGACCGTTTTTCGCTCTCTATTGTGCTGATGAATCAATACTTCTGACTTATT
CTCATTTATTCTACATAAAATTGCAACACTTAACAAAGAATTTTGAAGTGTTTTAGACGAAGATATTTAA
TGTTTAAATCTTCACTTTCATTGTAATAAAGTTCCATTTCTATTAGAAATCGCAAATTGATTTGTTTTCAAG
AATCTTTGTCAATAATTACATGAAATATTTCATGTACGCAGACTTTTTAAAAAATCCTTGACAAAAGATA
TGTTAGTTATGTCGATCAAACGAAAAAATCACGAGATATTATTACATGCCTGTAAACATGAATTATCTAA
ATGATCGTGAAGTGTTCCAAGAACTTGTTCAACTTAAAGTAAAATAAGTGATTATTATTTTTAAAAAAAT
TTAATGACATTCATTTATATTTCACATAAACATTTCTGTAATCAGTACGCATTTAATTTCTTCTGCAAATGG
TAAAATTTAAAAAATGAAATGTTTCTTTTTAATGGCTCATTAATGTTGTTATAATTGGTGGAAACAGATGG
ATCGAACATGCAGAATCCACGTGTACGCGTTGTACCTGTTTGCATTCGATGTGTATAAATATCGACTTCT
GCTCGTGGTAAATC**A**

> BmPI-1
TAAAACTTGTTTATTCTCATTTATAATAAAAATTAACTGCTATAATTATTTATAACTCGCATACAAATATTA
TTAACGATATTTTTAAATTAATCGGCCATAGTTAAAAAACTGTATTAAGAAAATTGAGTTTTTGTTATTAA
TGTTTCTGGCACGAAATTTTAAATAGTAAAATTAAACGTATGATTGAATTAATAATATTTTTCTCCATTTAC
AAATATTTTTAAACAA*AGATTGAAAAATAT*TTTAAAAAAAAGAGGAAAAACAAATTCGCGCCATCTAGT
GGTCATTTATTTCGATACAATTATTTAATTCTGTTATATAATTATTTATTTTTTATAATGTAATTATAAACTAT
TAACCTATAAATCGTCATTATTATTAATTTATACTGTATTTTTATTGAATGAGAATGAAAAATTGGAAATAA
TAAGAAATACACGTAATTGTTTAATTTTTTTTTCTTGATTAAAACTTCGACATTAAGATAACTGTATGTTTT
GAAACAAACTTCGCAAACATGCTTAGTAGTCATCAAAACATGAAATTTAACAGATACAATTCGAGAAA
CAATACGCAGCATGATCTAAAAGCAATTTACTTATGAAAAATTGTACATTGTGATGATTTGATGTATCTG
ATGATGGATTTTTATCAAGAATGTTTGCTTGGAGTTAAAATGATAGCATTGATTTGAAAATAAGAAGTTG
TTATTGCAGCAGATACGTCCATCTTTTGGTGCTATTTTCCATTACGTCACTTAAATAAATATTAAAAAATA
AAAGTTTTAAAAGAATGTTTACAGTTTAAAACTTTCATAAACTACGGTGTATGTTATTTAATGATTTTGCT
GAACTTACTGCATCGAGAATAATCTTAAAGCACTCCCAACTCAATTGTTACACGACAAATACGTGTTTA
CATATATTTTTATTATTTTCTGGGTAATTATTTTTATATTTTTTGAAGAATTAAACACGGATAGCTTTTGACA
TTTTAC**A**

## Reference

1 Messeguer, X.; Escudero, R.; Farré, D.; Nuñez, O.; Martínez, J.; Albà, M. PROMO: Detection of known transcription regulatory elements using species-tailored searches. *Bioinformatics* 2002, 18, 333–334.
